# Role of naringin in the treatment of atherosclerosis

**DOI:** 10.3389/fphar.2024.1451445

**Published:** 2024-09-06

**Authors:** Yan Lu, De-Hong Li, Ji-Mei Xu, Sheng Zhou

**Affiliations:** ^1^ Department of Clinical Laboratory, Gansu Provincial Hospital, Lanzhou, China; ^2^ Department of General Surgery, Gansu Provincial Hospital, Lanzhou, China; ^3^ Department of Radiology, Gansu Provincial Hospital, Lanzhou, China

**Keywords:** naringin, atherosclerosis, vascular smooth muscle cell, endothelial cell, inflammation, antioxidant

## Abstract

Atherosclerosis (AS) is a major pathological basis of coronary heart disease. However, the currently available medications are unable to effectively reduce the incidence of cardiovascular events in the majority of patients with AS. Therefore, naringin has been attracting considerable attention owing to its anti-AS effects. Naringin can inhibit the growth, proliferation, invasion, and migration of vascular smooth muscle cells, ameliorate endothelial cell inflammation and apoptosis, lower blood pressure, halt the cell cycle at the G1 phase, and impede growth via its antioxidant and free radical scavenging effects. These activities suggest the potential anti-AS effects of naringin. In this review article, we comprehensively summarized the latest findings on the anti-AS effects of naringin and their underlying mechanisms, providing a crucial reference for future research on the anti-AS potential of this agent.

## 1 Introduction

Atherosclerosis (AS) is a chronic inflammatory cardiovascular disease characterized by lipid deposition in the vascular wall and immune cell recruitment. This condition is a major risk factor for chronic heart disease (Liu et al., 2023). According to World Health Organization data, coronary heart disease accounts for seven million deaths worldwide annually (Liu et al., 2023). The main strategies for the prevention and treatment of arteriosclerosis include a balanced diet (Przybylska and Tokarczyk, 2022), body mass management (Hwang et al., 2023), smoking cessation and limited consumption of alcohol, regular physical activity (Xu et al., 2022), control of blood pressure, regulation of serum sugar and blood lipid levels, and anti-platelet therapy (Virani et al., 2023). However, the use of currently available medication can effectively reduce cardiovascular events in only 40% of patients with AS (Parsamanesh et al., 2019). An increasing number of studies focus on traditional Chinese medicine and its active ingredients due to their lipid-lowering and anti-AS effects.

Naringin is a flavonoid abundantly found in fruits and vegetables (particularly grapefruit and tomatoes) (Memariani et al., 2021; Viswanatha et al., 2017), which exists in two forms (i.e., naringenin and naringin). Naringenin is formed through rapid glycosylation of naringin by the liver enzyme naringinase. It has been established that the sour and bitter taste of citrus fruits is attributed to naringin rather than naringenin. Most clinical studies have shown that the main pharmacological effects of grapefruit are produced by naringin (Adetunji et al., 2023; Bailey et al., 2007). Moreover, evidence indicates that, in addition to the liver enzyme naringinase, intestinal bacteria possess the capability to hydrolyze naringin into naringenin. This process has the potential to generate hypoglycemic and hypolipidemic effects (Xulu and Oroma Owira, 2012). Due to the inhibitory effects of naringin on liver enzymes (cytochrome P450 enzymes), the consumption of grapefruit juice can reduce or increase the concentration of drugs that are metabolized in the liver, thereby altering their pharmacokinetics and potentially leading to toxicity (Heidary Moghaddam et al., 2020). Naringin exhibits various pharmacological activities, namely, antioxidant (Balachandran et al., 2023), anti-inflammatory (Zhang et al., 2022), anti-apoptotic (Zhao et al., 2020), anti-ulcer (Wang et al., 2023), and anti-osteoporotic (Pi et al., 2023), among others. Thus, increasing attention has been focused on the anti-AS effects of naringin. Naringin inhibits vascular smooth muscle cell (VSMC) proliferation (Lee et al., 2009) and ameliorates endothelial cell inflammation and apoptosis (Zhao et al., 2020) by exerting antioxidant effects and scavenging free radicals (Zhang et al., 2022; Amini et al., 2022). In this review, we summarized the latest available information on the anti-AS effects of naringin and their underlying mechanisms, providing a reference for future research on the anti-atherosclerotic properties of naringin.

Hypertension (Global Cardiovascular Risk et al., 2023), dyslipidemia (Global Cardiovascular Risk et al., 2023; Capra et al., 2023), endothelial cell injury (Li et al., 2014; Li et al., 2017), and smooth muscle proliferation are pivotal factors in the pathogenesis of AS (Feng and Xu, 2023). Naringin exerts anti-atherosclerotic effects by intervening in hypertension, ameliorating lipid disorders, safeguarding against endothelial cell damage, inhibiting smooth muscle proliferation, regulating the cell cycle, and modulating other mechanisms. The underlying mechanism may involve antioxidation, anti-inflammation, regulation of autophagy, and modulation of other pathways.

## 2 Naringin prevents AS by lowering blood pressure

**FIGURE 1:**
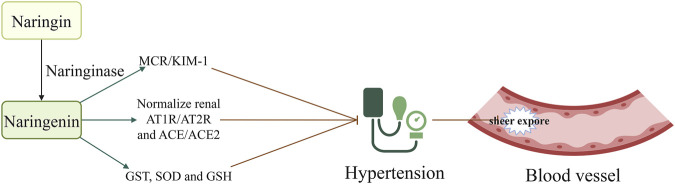
Naringin decreased sheer stress injury by lowering blood pressure; this effect was mediated by the attenuation of MCR/KIM1, and normalization of AT1R/AT2R and ACE/ACE2 ratios. Naringin is rapidly metabolized by the liver enzyme naringinase to produce naringenin. Naringenin decreased blood pressure and reduced vascular shear stress injury. Consequently, it decreased the occurrence of tissue atherosclerosis by adjusting the MCR/KIM1, AT1R/AT2R, and ACE/ACE2 ratios, and increasing the levels of GST, SOD, and GSH. Abbreviations: AT1R, angiotensin II type 1 receptor; AT2R, angiotensin II receptor type 2; ACE: angiotensin converting enzyme; GSH, reduced glutathione; GST, glutathione S-transferase; KIM, kidney injury molecule; MCR, mineralocorticoid receptor; SOD, superoxide dismutase.

Hypertension is associated with up to 13.5% of all deaths annually worldwide, and is the leading risk factor for cardiovascular disease, including AS (Global Cardiovascular Risk et al., 2023). Strict control of blood pressure significantly decreases the rates of cardiovascular events and all-cause mortality (Yntema et al., 2023; Virani et al., 2021). Findings have demonstrated the significant blood pressure-lowering effects of naringin in hypertensive rats with renal artery occlusion, potentially through the inhibition of oxidative stress (Visnagri et al., 2015). Additionally, naringin reduces systolic blood pressure in rats fed a high-carbohydrate and -fat diet, and improves both vascular and ventricular diastolic dysfunction (Alam et al., 2013). Its antihypertensive effects may be associated with the reduction of inflammatory cell infiltration, oxidative stress, plasma lipid concentrations, and improvement of hepatic mitochondrial function in rats (Alam et al., 2013). In another study, naringenin (a metabolite of naringin) significantly decreased cerebral glutathione S-transferase (GST) and superoxide dismutase (SOD) activity as well as the levels of glutathione (GSH) in rats with hypertension induced by oral administration of L-N^G^-Nitro arginine methyl ester (L-NAME) (Oyagbemi et al., 2020). Moreover, it restored the activity of renal catalase, SOD, GST, glutathione peroxidase (GPX), and glutathione reductase (GSR). Additionally, naringenin significantly decreased the expression of renal angiotensin-converting enzyme (ACE) and mineralocorticoid receptor (MCR) (Oyagbemi et al., 2020). These observations indicate that the hypotensive effect of naringenin is mediated by modulation of the MCR/ACE/kidney injury molecule 1 (MCR/ACE/KIM1) signaling pathway, and by its antioxidative properties (Oyagbemi et al., 2020). Other studies have demonstrated that naringin reduces angiotensin II receptor type 2 (AT2R) mRNA expression in the medulla and the ratio of AT1R/AT2R, while increasing ACE expression and the ACE/ACE2 ratio in the cortex and medulla. These findings propose a potential mechanism underlying the beneficial effects of naringenin on hypertensive nephropathy (Wang et al., 2019), as it restores the ACE/ACE2 imbalance and normalizes the renal AGTR1/AGTR2 protein ratio in hypertensive rats (Wang et al., 2019). However, contrasting evidence from the two-kidney, one-clip (2K1C) hypertension rat model suggests that naringenin does not affect blood pressure. This indicates that its antihypertensive effects might be specific to different models of hypertension (Wang et al., 2019) (Figure 1; Table 1). Naringin has demonstrated efficacy in reducing blood pressure in hypertensive rats with renal artery occlusion-induced renovascular dysfunction (Visnagri et al., 2015) and in rats fed a high-carbohydrate and high-fat diet (Alam et al., 2013). However, in two-kidney one-clip (2K1C) (Wang et al., 2019) and NG-nitro-L-arginine methyl ester (L-NAME)-induced hypertensive left ventricular hypertrophy models (Gao et al., 2018), naringin was found to reduce ACE 1 expression; however, it did not significantly affect blood pressure. We had initially attributed the inconsistent impact of naringin on blood pressure in hypertensive animal models to variations in dosage and administration methods. However, a detailed review of the literature showed that all four modes of administration involved intra-intestinal delivery with similar dose ranges. This controlled for the potential influence of drug dosage and administration routes on the antihypertensive efficacy of naringin. We believe that the inconsistent effects of naringin on blood pressure in hypertensive rats may be attributed to the pathogenesis of hypertension and factors related to the animal model. In rats with L-NAME-induced hypertension, a reduction in nitric oxide (NO) is primarily involved; the mechanism underlying hypertension induced by both the 2K1C model and renal artery obstruction appears similar. However, the degree of renal artery occlusion may vary. In both 2K1C and renal artery obstruction rat models, naringin decreased ACE1 expression; this was possibly caused by inconsistent reductions in ACE1 levels within each model. Blood pressure therefore remained unaffected despite an improvement in the kidney lesions. The effects of naringin on blood pressure in hypertensive rat models warrant further investigation.

**TABLE 1:** Main opinions regarding the mode of action of naringin in the treatment of atherosclerosis.

	Reference	Mode	Main opinion
1	Zhao and Zhao (2022)	HUVEC damage induced by TMAO	Naringin inhibited endothelial inflammation, oxidative stress, and degradation of tight junction proteins, such as ZO-2, OCLN, and vascular endothelial-cadherin. Furthermore, it promoted NO release, thereby restoring the functional and structural integrity of the endothelium and inactivating MAPK signaling.
2	Wang et al. (2020a)	ApoE^−/−^ mice fed a high-fat diet	Naringin increased the excretion of bile acids and neutral sterols mainly by modulating the abundance of bile salt hydrolase- and 7α-dehydroxylase-producing bacteria. This led to the promotion of bile acid synthesis from cholesterol by upregulating CYP7A1 via suppression of the FXR/FGF15 pathway. In addition, naringin facilitated reverse cholesterol transport by downregulating PCSK9/IDOL.
3	Chanet et al. (2012)	Wild-type mice fed a high-fat/high-cholesterol diet, ApoE-deficient mice fed a semisynthetic diet	Naringin supplementation reduced plaque progression and the levels of plasma non-high-density lipoprotein cholesterol. It limited atherosclerosis progression by preventing immune cell adhesion and infiltration in the intima of the vascular wall, as well as smooth muscle cell proliferation.
4	Lee et al. (2008)	VSMCs	Naringin inhibited growth and induced cell cycle arrest at the G1 phase (mediated by induction of p53-independent p21WAF1 expression), downregulated the expression of CCNs and CDKs by inhibiting ERK function and p21WAF1 expression, and increased the activation of both Ras and Raf by the Ras/Raf/ERK pathway.
5	Lee et al. (2009)	VSMCs	Naringin impeded the TNF-α-induced expression of MMP9 in VSMCs, as well as invasion and migration. It also inhibited the TNF-α-mediated release of IL6 and IL8. Following administration to VSMCs in the presence of TNF-α, naringin did not affect cell growth and apoptosis. Furthermore, naringin reduced the transcriptional activity of AP-1 and NF-κB, and blocked the PI3K/AKT/mTOR/p70S6K pathway in TNF-α-induced VSMCs. These findings suggest that naringin repressed the PI3K/AKT/mTOR/p70S6K pathway in TNF-α-induced VSMCs, as well as invasion and migration. This was followed by suppression of MMP9 expression through transcription factors NF-κB and AP-1.
6	Zhao et al. (2020)	ox-LDL-induced HUVECs	Pretreatment with naringin inhibited ox-LDL-induced cell injury and apoptosis, and restored the integrity of the endothelial barrier by preventing vascular endothelial-cadherin disassembly and F-actin remodeling in HUVECs. Additionally, treatment with naringin downregulated the expression of pro-inflammatory factors, such as IL1β, IL6, and IL18, in these cells. Naringin-induced YAP downregulation was restored, as demonstrated by the attenuation of the cytoprotective effect of naringin on ox-LDL-induced endothelial cell injury and apoptosis by YAP-shRNA.
7	Pengnet et al. (2019)	Hypercholesterolaemic rats	In hypercholesterolaemic rats, treatment with naringin enhanced aortic NO levels, restored endothelium-dependent responses to acetylcholine, and reduced the levels of O_2_^−^, LOX-1, NOX subunits (p47^phox^, NOX2, and NOX4), and iNOS, as well as the expression of oxidative damage markers (3-nitrotyrosine and 4-hydroxynonenal) in aortic tissues.
8	Mao et al. (2017)	Thoracic aortas vascular ring	Naringin inhibited the expression of VEGF, CRP, JNK2, p38, and the MAPK pathway. These effects resulted in the decrease of NO synthesis, VEGF expression, and endothelial adhesion factor expression.
9	Bi et al. (2016)	LPS-induced damage in HUVECs	Naringin enhanced the survival rate of HUVECs, concurrently reducing LPS-induced ROS generation and intracellular Ca (2+) levels. Moreover, it exhibited a significant reduction in cytochrome C release from mitochondria to the cytosol. This phytochemical agent downregulated the protein or mRNA levels of IL1, IL6, TNF-α, VCAM1, ICAM1, NF-κB, AP-1, cleaved-CASP3/7/9, p53, BAK, and BAX, while upregulating the expression of BCL-xl and BCL2 to suppress inflammation and apoptosis. Additionally, naringin demonstrated a notable inhibitory effect on the phosphorylation levels of JNK, ERK, and p38 MAPK.
10	Hsueh et al. (2016)	HUVECs	Naringin impeded the adhesion of THP-1 monocytes to TNF-α-stimulated HUVECs, and suppressed the expression of cell adhesion molecules (VCAM-1, ICAM1, and SELE) induced by TNF-α. Furthermore, naringin inhibited the mRNA and protein levels of chemokines, including fractalkine/CX3CL1, MCP-1, and RANTES, stimulated by TNF-α. Significantly, naringin obstructed the nuclear translocation of NF-κB induced by TNF-α, which arises from the inhibited phosphorylation of IKKα/β, IκB-α, and NF-κB.
11	Li et al. (2014)	TNF-α induced HUVECs	Naringin impeded the generation of ROS and the overexpression of NOX4 and p22^phox^ triggered by TNF-α. Furthermore, naringin obstructed the TNF-α induced overexpression of ICAM1 and VCAM-1 (mRNA and protein). Lastly, naringin also suppressed the activation of the NF-κB and PI3K/Akt signaling pathways.
12	Xulu and Oroma Owira (2012)	Diabetes rats induced by streptozotocin intraperitoneally	Naringin reduced the levels of blood LDL, elevated HDL, and decreased hepatic total cholesterol and triglycerides in diabetic rats. It inhibited hepatic HMGCR and Acyl-CoA: cholesterol acyltransferase activities. Naringin modulated the plasma low-density lipoprotein to high-density lipoprotein ratio.
13	Chanet et al. (2012)	Mouse models of hypercholesterolemia, wild-type mice fed a high-fat/high-cholesterol diet and ApoE-deficient mice fed a semisynthetic diet	Naringin decreased the plasma levels of non-high-density lipoprotein cholesterol and modulated biomarkers of endothelial dysfunction. Consequently, the alterations in gene expression induced by naringin imply a restricted progression of atherosclerosis by inhibiting immune cell adhesion and infiltration within the intima of vascular walls, as well as suppressing the proliferation of smooth muscle cells. Additionally, naringenin efficiently mitigated monocyte adhesion to endothelial cells and smooth muscle cell proliferation.
14	Jeon et al. (2001)	Male rabbits were fed a high-cholesterol diet (0.5%, w/w) and naringin (0.05%, w/w) or lovastatin (0.03%) supplemented high-cholesterol diet.	Naringin significantly increased the concentration of plasma vitamin E, and upregulated the mRNA expression of SOD, catalase, and GSH-Px. These results indicate that naringin plays an important role in regulating antioxidative capacities.
15	Choe et al. (2001)	New Zealand white rabbits were fed a 0.25% cholesterol diet	Naringin effectively reduced the size of fatty streak lesions in the thoracic aorta, inhibited subintimal foam cell infiltration, and suppressed hypercholesterolemia-induced ICAM1 expression on endothelial cells. Furthermore, it demonstrated potent protection against hypercholesterolemia-induced fatty liver and elevations in the levels of liver enzymes.
16	Lee et al. (2009)	TNF-α-induced invasion and migration of VSMCs	In VSMCs, naringin exhibited a suppressive effect on TNF-α-induced MMP9 expression, inhibited invasion and migration, and reduced the release of IL6 and IL8 mediated by TNF-α. However, it did not influence cell growth and apoptosis. Furthermore, in additional experiments, naringin demonstrated the ability to reduce the transcriptional activity of AP-1 and NF-κB, and inhibited the PI3K/AKT/mTOR/p70S6K pathway.
14	Wu et al. (2021)	Monocrotaline administration (60 mg/kg) was delivered for the induction of PAH in rats, HUVECs	Naringin significantly inhibited endothelial-to-mesenchymal transition and alleviated PAH progression induced by treatment with TGFβ1, enhanced endothelial marker expression, and inhibited the activation of the ERK and NF-κB signaling pathways.
17	Shangguan et al. (2017)	Vascular endothelial cells	Naringin markedly stimulated vascular endothelial cell proliferation, significantly inhibited serum starvation-induced apoptosis in endothelial cells, and suppressed GRP78, CHOP, CASP12, and cytochrome C protein expression. Additionally, it reduced the mitochondrial membrane potential and the activities of CASP3 and CASP9. Naringin also potently inhibited EDN1, while enhancing NO synthesis. Assessment of the distal femoral microvascular density revealed that the naringin treatment group exhibited a significantly higher number of microvessels compared with the ovariectomized group, as well as a positive correlation between microvascular density and bone mineral density.

Abbreviations: Acyl-CoA, acyl-coenzyme A; AKT, protein kinase B; AP-1, activator protein-1; ApoE^−/−^, apolipoprotein E^−/−^; BAK, BCL2 antagonist/killer; BAX, BCL2 associated X; BCL-xL, BCL-extra large; CASP, caspase; CDKs, cyclin-dependent kinases; CCNs, cyclins; CHOP, C/EBP, homologous protein; CRP, C-reactive protein; CX3CL1, C-X3-C motif chemokine ligand 1; CYP7A1, cytochrome P450 family 7 subfamily A member 1; EDN1, endothelin 1; ERK, extracellular signal-regulated kinase; FXR/FGF15, farnesoid X receptor/fibroblast growth factor 15; GSH-Px, glutathione peroxidase; GRP78, glucose-regulated protein, 78 kDa; HDL, high-density lipoprotein; HMGCR, 3-hydroxy-3-methylglutaryl CoA reductase; HUVECs, human umbilical vein endothelial cells; ICAM1, intercellular adhesion molecule 1; IDOL, E3 ubiquitin ligase-inducible degrader of the low density lipoprotein receptor; IL, interleukin; iNOS, inducible nitric oxide synthase; IKKα/β, IκB kinase α/β; JNK, JUN N-terminal kinase; LDL, low-density lipoprotein; LOX-1, lectin-like oxidized low-density lipoprotein receptor-1; LPS, lipopolysaccharide; MAPK, mitogen-activated protein kinase; MCP-1, monocyte chemoattractant protein-1; MMP9, matrix metalloproteinase 9; mTOR, mechanistic target of rapamycin kinase; NF-κB, nuclear factor-kappa B; NO, nitric oxide; NOX, NADPH, oxidase; O_2_^−^, superoxide anion; OCLN, occludin; ox-LDL, oxidized-low-density lipoprotein; PAH, pulmonary arterial hypertension; PCSK9, proprotein convertase subtilisin/kexin type 9; PI3K, phosphatidylinositol 3 kinase; RANTES, regulated upon activation, normally T-expressed, and presumably secreted; ROS, reactive oxygen species; SELE, selectin E; SOD, superoxide dismutase; TGFβ1, transforming growth factor β1; TMAO, trimethylamine-N-oxide; TNF-α, tumor necrosis factor-alpha; VCAM1, vascular cell adhesion molecule 1; VEGF, vascular endothelial growth factor; VSMCs, vascular smooth muscle cells; WAF1, wild-type p53 activated fragment-1; YAP, yes-associated protein; ZO-2, zona occludens-2.

## 3 Naringin prevents AS by ameliorating dyslipidemia

Dyslipidemia is widely recognized as an important risk factor associated with the morbidity and mortality caused by AS (Yu et al., 2022; Hong et al., 2022; Capra et al., 2023). Dyslipidemia is a metabolic disorder characterized by elevated levels of serum cholesterol, triglycerides, low-density lipoprotein, and very-low-density lipoprotein, as well as decreased levels of high-density lipoprotein cholesterol (Hong et al., 2022). Increasing evidence suggests that naringin exerts inhibitory effects on the progression of AS by ameliorating dyslipidemia (Pu et al., 2012). Naringin attenuates foam cell infiltration into plaques and reduces lesion areas in the aortic sinus (Li et al., 2014). Intragastric administration of naringin significantly reduced total cholesterol and triglyceride levels, while increasing high-density lipoprotein cholesterol levels in mice fed a high-fat diet (Yu et al., 2022). In type I diabetic rats, naringin did not significantly alter hyperglycemia; however, it effectively improved atherogenic dyslipidemia by increasing the levels of high-density lipoprotein cholesterol (Xulu and Oroma Owira, 2012). Moreover, in rabbits fed a 0.25% cholesterol diet, naringin reduced serum cholesterol levels and demonstrated remarkable efficacy in reducing the area of fatty streaks in the thoracic aorta and subintimal foam cell infiltration (Choe et al., 2001). Additionally, naringin inhibited the hypercholesterolemia-induced expression of intercellular adhesion molecule 1 (ICAM1) on endothelial cells, suggesting that the suppression of ICAM1 contributes to its anti-atherogenic effect (Choe et al., 2001).

Non-target metabolomics analysis showed that naringin modulates the hepatic levels of cholesterol derivatives and bile acids (Wang F. et al., 2020) by inhibiting 3-hydroxy-3-methylglutaryl-CoA reductase (HMGCR) and acyl-coenzyme cholesterol acyltransferase (ACCA), respectively. Further investigation suggested that the gut microbiota-liver-cholesterol axis may represent the primary potential pathway through which naringin exerts its anti-atherosclerotic effects. Naringin alters the abundance of bacteria producing bile salt hydrolase and 7α-dehydroxylase, thereby promoting bile acid synthesis from cholesterol. This is achieved by upregulating cholesterol 7α-hydroxylase (cytochrome P450 family 7 subfamily A member 1 [CYP7A1]) expression via suppression of the farnesoid X receptor/fibroblast growth factor 15 (FXR/FGF15) pathway (Wang F. et al., 2020). Through oral administration, naringin predominantly localizes in the intestine due to the high solubility of 7-O-nohesperidoside. It exerts its anti-atherosclerotic effects primarily by augmenting bile acid synthesis through modulation of the gut microbiota-FXR/FGF15-CYP7A1 pathway (Wang F. et al., 2020). Naringin modulates the composition of gut microbiota by reducing the relative abundance of *Bacteroides*, *Bifidobacterium*, and *Clostridium* genera, which are associated with bile acid metabolism, while increasing that of *Eubacterium* genus that possesses bile acid-hydrolyzing activity and reduces the levels of bile acids (Wang F. et al., 2020). The administration of naringin facilitates the accumulation of conjugated bile acids, including tauro-α/β-muricholic acid, while concurrently inhibiting the expression of FGF15 through the inactivation of FXR. The expression of CYP7A1, which encodes the rate-limiting enzyme in the biosynthesis of bile acids from cholesterol, was enhanced by downregulation of the FXR/FGF pathway (Wang F. et al., 2020). Modulation of gut microbiota remodeling by naringin (a natural polyphenolic compound) has a profound impact on cholesterol metabolism and AS. In addition, recent findings have shown that naringin supplementation increases the abundance of Bifidobacterium and Lachnospiraceae bacterium 28–4, while reducing that of Lachnospiraceae bacterium DW59 and Dubosiella newyorkensis. Naringin supplementation has also been found to alter the fecal metabolite profile by significantly promoting the production of taurine, tyrosol, and thymol, which offers benefits in atherosclerosis by reducing oxidative stress, inflammation, hyperlipidemia, and lipid lesions in the aortic intima (Zoubdane et al., 2024; Yu et al., 2016).

**FIGURE 2:**
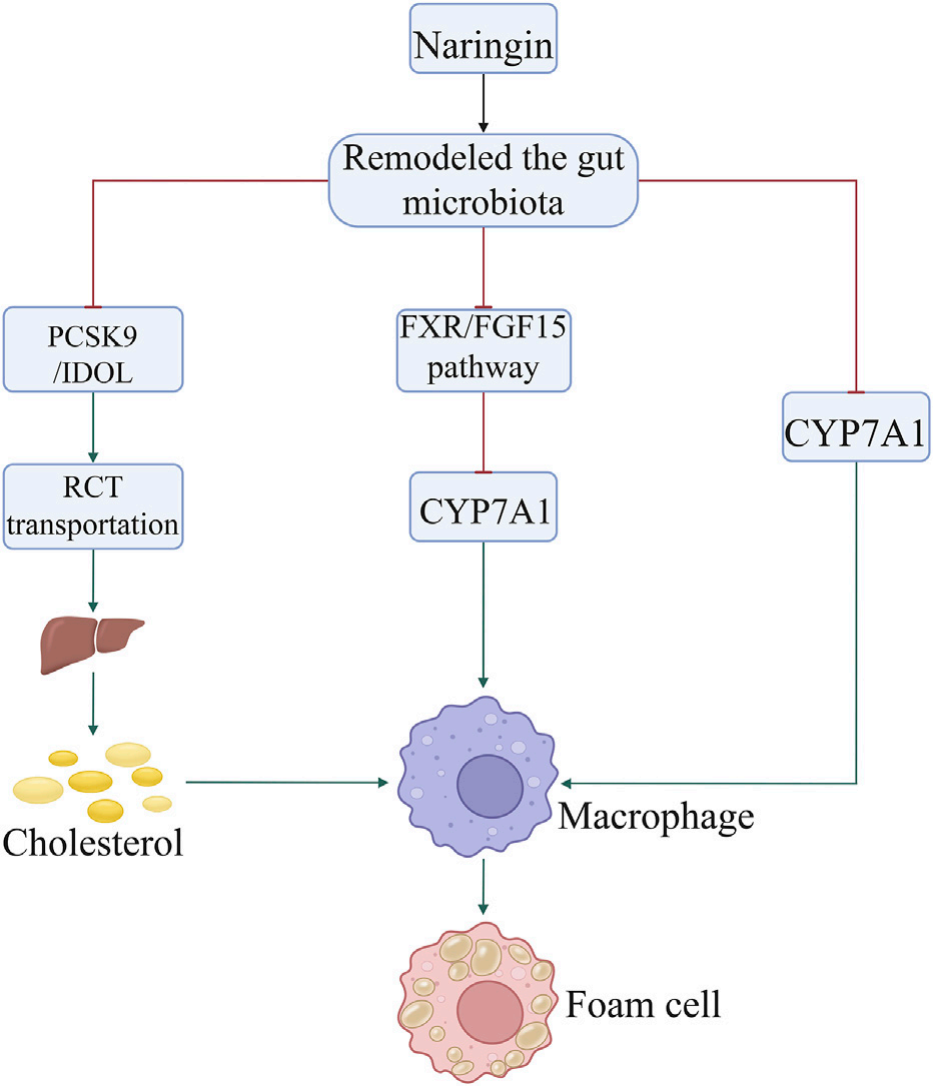
Naringin inhibited the formation of foam cells by decreasing the uptake of cholesterol by macrophages through regulation of the gut microbiota. Naringin suppressed the expression of PCSK9, IDOL, FXR/FGF15 pathway, and HMGCR by gut microbiota remodeling, thereby promoting RCT to the liver from peripheral tissues. Abbreviations: CYP7A1, cholesterol 7α-hydroxylase; FXR/FGF15, farnesoid X receptor/fibroblast growth factor 15; HMGCR, 3-hydroxy-3-methylglutaryl-CoA reductase; IDOL, inducible degrader of low-density lipoprotein receptor; PCSK, proprotein convertase subtilisin/kexin type; RCT, reverse cholesterol transport.

In addition, naringin suppressed the expression of proprotein convertase subtilisin/kexin type 9 (PCSK9) and an inducible degrader of low-density lipoprotein receptor (IDOL), thereby promoting reverse cholesterol transport to the liver from peripheral tissues. The excretion of cholesterol from the liver to the gallbladder was also enhanced, as was that via the enterohepatic circulation. These mechanisms collectively decrease the cholesterol levels, thus alleviating AS. Notably, the expression of genes in the FXR/FGF15 pathway, particularly CYP7A1, was significantly inhibited by gut microbiota remodeling. Therefore, modulation of cholesterol metabolism by gut microbiota remodeling may be implicated in the effect of naringin on AS (Figure 2; Table 1).

Furthermore, naringin promotes reverse cholesterol transport by downregulating the expression of PCSK9/IDOL (Wang F. et al., 2020). Clinical investigations revealed that naringin significantly reduces the body mass index, total cholesterol, and low-density lipoprotein cholesterol, whereas it concurrently elevates the levels of adiponectin in adult patients diagnosed with dyslipidemia. These findings suggest that naringin holds potential as a therapeutic agent in the management of metabolic disorders (Barajas-Vega et al., 2022). The findings were further validated by the inclusion of bergamot juice extract, which contains naringin as one of its primary bioactive constituents. Supplementation with this extract has been shown to effectively lower blood lipid levels, and thereby offer potential benefits for individuals with dyslipidemia (Toth et al., 2015).

## 4 Naringin attenuates progression of AS by safeguarding endothelial dysfunction

Naringin enhances the survival rate of human umbilical vein endothelial cells (HUVECs) and preserves the functional and structural integrity of the endothelium, which plays a crucial role in inhibiting the advancement of AS. Thus, the preventive effect of naringin on endothelial dysfunction could potentially delay the progression of AS (Li et al., 2014). Pretreatment with naringin attenuates endothelial inflammation, reduces oxidative stress, enhances NO release, and inhibits the degradation of zona occludens 2 (ZO-2), occludin (OCLN), and vascular endothelial-cadherin. These effects preserve the functional and structural integrity of the endothelium (Zhao and Zhao, 2022). It has been demonstrated that the therapeutic effects of naringin are mediated through the inhibition of trimethylamine-N-oxide (TMAO)-stimulated mitogen-activated protein kinase (MAPK) signaling in HUVECs (Zhao and Zhao, 2022). In another investigation, naringin augmented the survival rate of HUVECs and mitigated the elevations in the levels of reactive oxygen species (ROS) and intracellular Ca^2+^ induced by lipopolysaccharide in comparison to the control group (Bi et al., 2016). Moreover, naringin impedes cytochrome C release from mitochondria to cytosol, notably represses the protein or mRNA expression of interleukin 1 (IL1), IL6, tumor necrosis factor-alpha (TNF-α), vascular cell adhesion molecule 1 (VCAM1), ICAM1, nuclear factor-kappa B (NF-κB), activator protein-1 (AP-1), cleaved caspase 3/7/9 (CASP3/7/9), p53, and BCL2 associated X (BAX), and enhances the expression of BCL-extra large (BCL-xl) and BCL2 to curtail inflammation and apoptosis (Zhao and Zhao, 2022). Additionally, naringin robustly inhibits the phosphorylation levels of JUN N-terminal kinase (JNK), extracellular signal-regulated kinase (ERK), and p38 MAPK (Bi et al., 2016), and reduces the expression of cell adhesion molecules (VCAM1, ICAM1, and selectin E [SELE]), which are associated with the adhesion of THP-1 monocytes to HUVECs induced by TNF-α (Li et al., 2014). In addition, naringin markedly reduced the mRNA and protein levels of chemokines, such as fractalkine/C-X3-C motif chemokine ligand 1 (fractalkine/CX3CL1), and monocyte chemoattractant protein-1 (MCP-1), and regulated the activation of normal T cell expressed and secreted, induced by TNF-α (Hsueh et al., 2016). This effect is attributed to the potent inhibition of TNF-α-induced nuclear translocation of NF-κB by naringin, which is mediated by the suppression of IκB kinase α/β (IKKα/β), IκB-α, and NF-κB phosphorylation (Hsueh et al., 2016). These findings indicate that naringin exerts an anti-atherosclerotic effect by modulating the expression of cell adhesion molecules and chemokines. This is achieved by inhibiting the activation of the IKK/NF-κB signaling pathway induced by TNF-α (Hsueh et al., 2016). Naringin inhibits the production of ROS and the overexpression of NADPH oxidase 4 (NOX4) and p22^phox^ induced by TNF-α. Furthermore, it suppresses the activation of the NF-κB and phosphatidylinositol 3 kinase/protein kinase B (PI3K/AKT) signaling pathways. These findings suggest that naringin exerts preventive effects on HUVEC injury induced by alleviated oxidative stress and the inflammatory response. The potential mechanisms underlying these effects may involve inhibition of the NOX4 and NF-κB pathways, as well as activation of the PI3K/AKT pathway (Wang K. et al., 2020). Naringin inhibits the expression of vascular endothelial growth factor (VEGF), C-reactive protein (CRP), JNK2, p38, and NO, indicating that it reduces CRP expression, curtails the activity of JNK2 and p38 kinases, and represses the MAPK pathway. This leads to a decline in NO synthesis, VEGF levels, and endothelial adhesion factor expression (Pengnet et al., 2019). These observations suggest that naringin regulates the inactivation of NO and shields endothelial function from ROS through its potent antioxidant properties (Ikemura et al., 2012).

**FIGURE 3:**
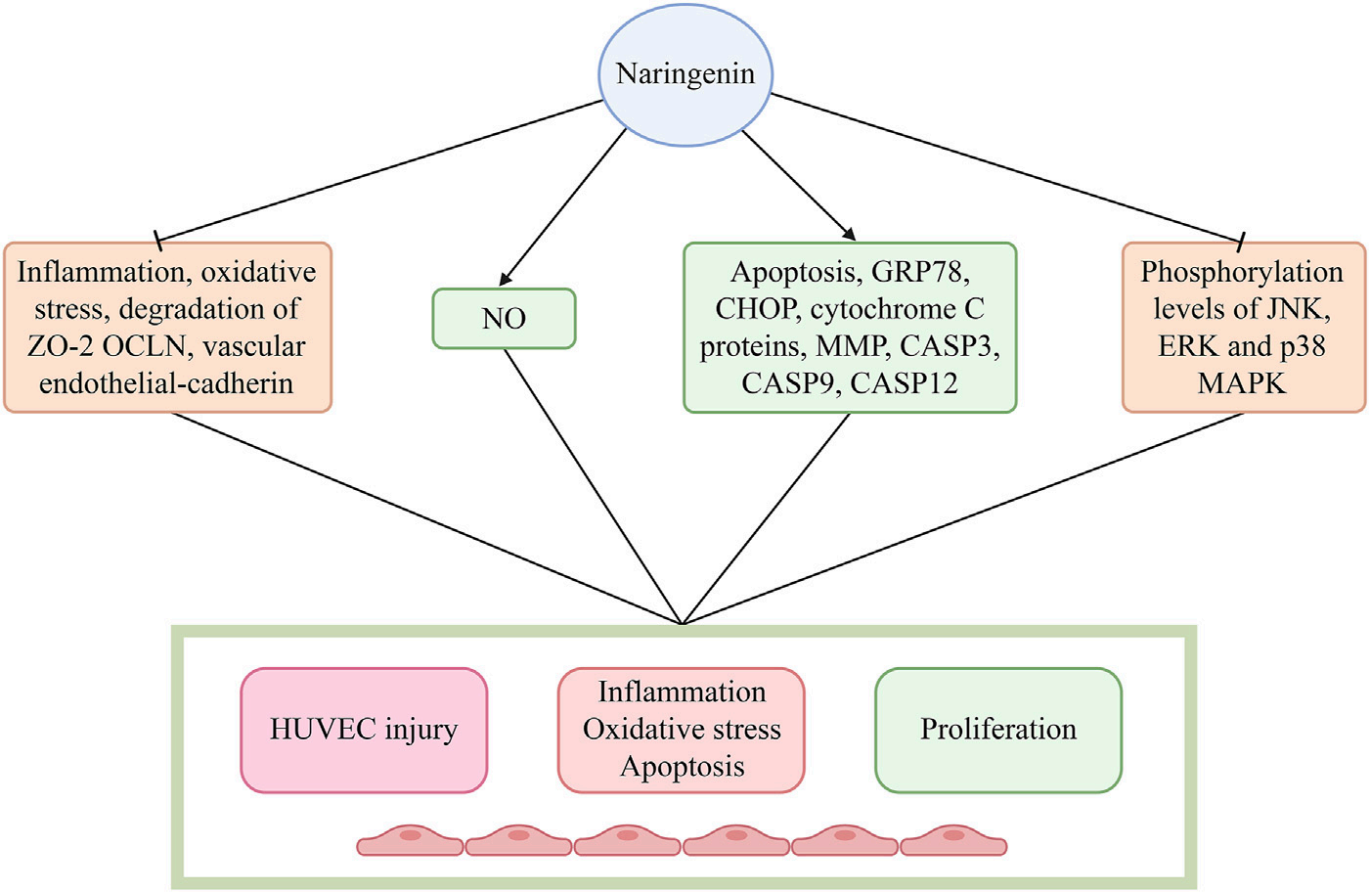
Naringin restored the functional and structural integrity of the endothelium by decreasing endothelial inflammation, reducing oxidative stress, increasing NO release, and inhibiting the degradation of zona occludens-2 (ZO-2), occludin (OCLN), and vascular endothelial-cadherin. Naringin obviously inhibited the phosphorylation of JNK, ERK, and p38 MAPK, and decreased the expression of cell adhesion molecules (VCAM-1, ICAM1, and SELE), which are related with the adhesion of THP-1 monocytes to HUVECs induced by TNF-α. Abbreviations: CASP3/9/12, caspase 3/9/12; CHOP, C/EBP homologous protein; ERK, extracellular signal-regulated kinase; GRP78, glucose-regulated protein, 78 kDa; HUVECs: human umbilical vein endothelial cells; ICAM1: intercellular cell adhesion molecule-1; JNK, JUN N-terminal kinase; MAPK, mitogen-activated protein kinase; NO, nitric oxide; SELE, selectin E; TNF-α, tumor necrosis factor-α; VCAM-1, vascular cell adhesion molecule-1.

Naringin also significantly reduced serum starvation-induced apoptosis in endothelial cells (Shangguan et al., 2017). Moreover, it promotes the proliferation of lung vascular endothelial cells in newborn rats, downregulates the expression of glucose-regulated protein, 78 kDa (GRP78), C/EBP homologous protein (CHOP), CASP12, and cytochrome C proteins, reduces the mitochondrial membrane potential, and decreases the activities of CASP3 and CASP9 (Shangguan et al., 2017). In addition, naringin exhibits dual functionality *in vitro* and *in vivo* by suppressing endothelin and enhancing NO synthesis. In summary, naringin exerts its anti-apoptotic effect on vascular endothelial cells by inhibiting the endoplasmic reticulum stress- and mitochondria-mediated pathways, thereby regulating endothelial cell function. Furthermore, naringin promotes angiogenesis, thus exerting its anti-osteoporotic effect (Shangguan et al., 2017) (Figure 3; Table 1).

## 5 Naringin inhibits the growth, proliferation, invasion, and migration of VSMCs, and induces cell cycle arrest at the G1 phase, demonstrating its potential utility in the prevention of AS

Naringin exerts a significant inhibitory effect on cell growth and triggers VSMCs in response to TNF-α. The inhibitory mechanisms of naringin on TNF-α-induced VSMC proliferation, invasion, and migration might be mediated by the downregulation of matrix metalloproteinase 9 (MMP9) and AKT phosphorylation, which acts as a downstream effector of PI3K (Lee et al., 2009). Besides, treatment with naringin reduced TNF-α-induced MMP9 secretion by decreasing the binding of transcription factors NF-kB and AP-1 to DNA (Lee et al., 2009). Naringin also inhibited TNF-α-induced AKT, mechanistic target of rapamycin kinase (mTOR), and p70S6K phosphorylation (Lee et al., 2009). These results corroborate the notion that naringin inhibits the PI3K/AKT/mTOR/p70S6K pathway (Lee et al., 2009), which could potentially explain the mechanisms underlying the inhibitory effects of naringin on TNF-α induced proliferation, invasion, and migration of VSMCs.

**FIGURE 4:**
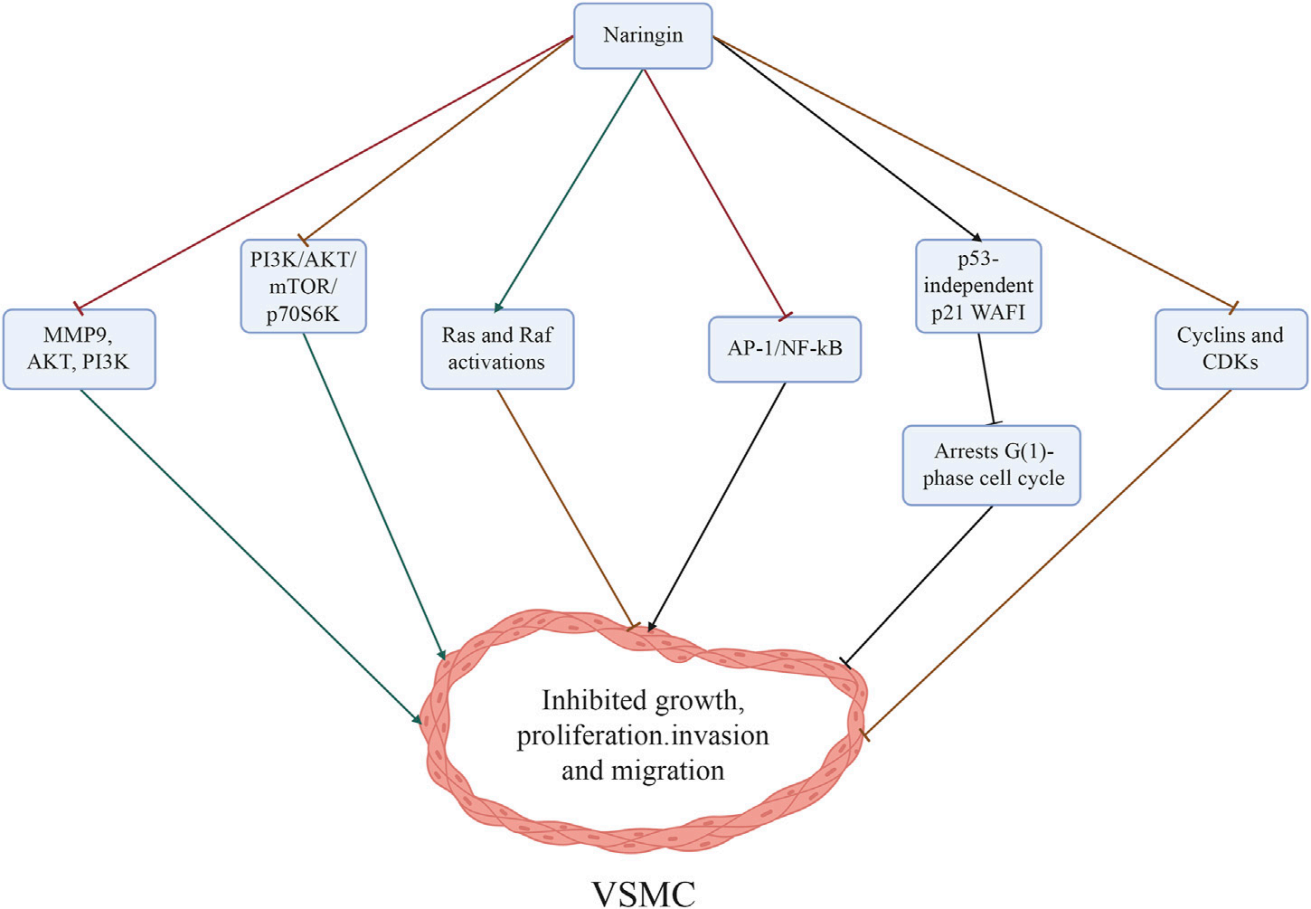
Mechanisms through which naringin inhibited the proliferation, invasion, and migration of VSMCs induced by TNF-α. The inhibitory effects of naringin on atherosclerosis were mediated by its ability to downregulate MMP9 and AKT phosphorylation (a downstream effector of PI3K), and reduce NF-κB activity, while also inhibiting the PI3K/AKT/mTOR/p70S6K axis. Naringin resulted in significant inhibition of growth and induced cell cycle arrest at the G1 phase by triggering p53-independent p21WAF1 induction, while concurrently downregulating the expression of cyclins (CCNs) and CDKs in VSMCs. In addition, blockage of ERK function repressed naringin-dependent p21WAF1 expression and concurrently enhanced the activation of Ras and Raf. Abbreviations: AKT, protein kinase B; AP-1, activator protein-1; CDKs, cyclin-dependent kinases; ERK, extracellular signal-regulated kinase; MMP, matrix metalloproteinases; mTOR, mammalian target of rapamycin; NF-κB, nuclear factor-κB; PI3K, phosphatidylinositol 3 kinase; VSMCs, vascular smooth muscle cells; WAF1, wild-type p53 activated fragment-1.

It was demonstrated that treatment with naringin significantly inhibited cell growth and induced cell cycle arrest at the G1 phase. These effects were mediated by the induction of p21 wild-type p53 activated fragment-1 (WAF1) expression independent of p53. Furthermore, it was found that naringin downregulated the expression of cyclins (CCNs) and cyclin-dependent kinases (CDKs) in VSMCs (Lee et al., 2008; Li et al., 2014). Inhibition of ERK function promotes naringin-dependent p21WAF1 expression, counteracts naringin-mediated suppression of cell proliferation, and reduces the levels of cell cycle proteins. Concurrently, treatment with naringin augments the activation of both Ras and Raf. Transfection of cells with dominant negative Ras (RasN17) and Raf (RafS621A) mutant genes impedes naringin-induced ERK activity and p21WAF1 expression. Attenuation of naringin-induced reduction in cell proliferation and cell cycle proteins was observed in the presence of RasN17 and RafS621A mutant genes. The involvement of the Ras/Raf/ERK pathway in p21WAF1 induction was demonstrated, resulting in a decline of CCND1/CDK4 and CCNE/CDK2 complexes and naringin-dependent inhibition of cell growth (Lee et al., 2008). Naringin (10–25 µM) exhibits inhibitory effects on TNF-α-induced MMP9 expression in VSMCs, as well as on invasion and migration. Additionally, it mitigates the TNF-α-mediated release of IL6 and IL8. However, treatment of VSMCs with naringin in the presence of TNF-α does not affect cell growth and apoptosis. In additional experiments, naringin suppressed the transcriptional activity of AP-1 and NF-κB, which are two crucial nuclear transcription factors implicated in MMP9 expression. Moreover, treatment with naringin inhibited the PI3K/AKT/mTOR/p70S6K pathway in TNF-α-induced VSMCs. These results suggest that naringin antagonizes the PI3K/AKT/mTOR/p70S6K pathway, thereby suppressing invasion and migration, and downregulating MMP9 expression through the transcription factors NF-κB and AP-1 in TNF-α-induced VSMCs (Lee et al., 2009) (Figure 4; Table 1).

## 6 Summary

In recent years, a growing body of evidence has corroborated the anti-atherosclerotic activity of naringin. These effects are attributable to its antioxidant capability, regulation of autophagy, amelioration of blood lipid disorders, inhibition of endothelial cell inflammation and vascular smooth muscle proliferation, reduction of the entry of low-density lipoprotein into macrophages, and suppression of foam cell formation (All views have been summarized in Table 1). However, most research has focused on fundamental inquiries, with relatively fewer clinical studies conducted thus far. Additional clinical trials are warranted to validate the therapeutic efficacy and potential adverse effects of naringin. In this context, the toxicity of naringin has been evaluated extensively; data from available studies have unequivocally demonstrated the toxic effects to be negligible. In their study, Li et al. (2014) found that naringin did not demonstrate any significant acute or chronic oral toxicity in SD rats. They also found the no-observed-adverse-effect-level in beagle dogs to be at least 500 mg/kg body weight per day, on oral administration for 3 and 6 consecutive months. These studies provide evidence of the favorable safety profile of naringin (Li et al., 2020). Such knowledge would assist in effectively utilizing naringin in clinical practice against AS. In its native state, naringin displays limited water solubility and weak efficacy. Thus, the application of nanoparticle formulation technology should be considered to alter the structure of naringin, with the aim to enhance its water solubility, bioavailability, and pharmacological activity (Dashputre et al., 2023). Additionally, a comprehensive assessment of its pharmacokinetic properties is crucial to fully investigate the potential benefits offered by this intriguing phytochemical.

## Figures and Tables

**Table:** 

**2K1C**	two-kidney one clip
**ACE**	angiotensin-converting enzyme
**AP-1**	activating protein-1
**CDKs**	cyclin-dependent kinases
**CX3CL1**	fractalkine
**GRP78**	glucose regulated protein 78
**HDL-C**	high-density lipoprotein cholesterol
**ICAM-1**	intercellular adhesion molecule-1
**IDOL**	low-density lipoprotein receptor
**IKK**	IκB kinase
**LDL**	low-density lipoprotein
**LPS**	lipopolysaccharide
**MCR**	mineralocorticoid receptor
**NF-kB**	nuclear factor-kB
**PCSK9**	proprotein convertase subtilisin/kexin type
**RCT**	reverse cholesterol transport
**SMC**	smooth muscle cell
**TNF-α**	tumor necrosis factor-alpha
**VEC**	vascular endothelial cell
**VLDL**	very-low-density lipoprotein
**WAF1**	wild-type p53 activated fragment-1
**AS**	atherosclerosis
**AKT**	protein kinase B
**ATR**	angiotensin receptor
**CHOP**	C/EBP-homologous protein
**ERK**	extracellular signal-regulated kinase
**GST**	glutathione S-transferase
**HUVEC**	human umbilical vein endothelial cell
**ICAM-1**	intercellular cell adhesion molecule-1
**MMP**	matrix metalloproteinase
**Kim**	kidney injury molecule
**L-NAME**	L-NG-nitro arginine methyl ester
**MAPK**	mitogen-activated protein kinase
**mTOR**	mammalian target of rapamycin
**NO**	Nitric oxide
**PI3**	phosphatidylinositol 3 kinase
**ROS**	reactive oxygen species
**SOD**	superoxide dismutase
**VCAM-1**	vascular cell adhesion molecule-1
**VEGF**	vascular endothelial growth factor
**VSMC**	vascular smooth muscle cell

## References

[B1] Adetunji J. A. Fasae K. D. Awe A. I. Paimo O. K. Adegoke A. M. Akintunde J. K. (2023). The protective roles of citrus flavonoids, naringenin, and naringin on endothelial cell dysfunction in diseases. Heliyon 9, e17166. 10.1016/j.heliyon.2023.e17166 37484296 PMC10361329

[B2] Alam M. A. Kauter K. Brown L. (2013). Naringin improves diet-induced cardiovascular dysfunction and obesity in high carbohydrate, high fat diet-fed rats. Nutrients 5, 637–650. 10.3390/nu5030637 23446977 PMC3705310

[B3] Amini N. Maleki M. Badavi M. (2022). Nephroprotective activity of naringin against chemical-induced toxicity and renal ischemia/reperfusion injury: a review. Avicenna J. Phytomed. 12, 357–370. 10.22038/AJP.2022.19620 35782769 PMC9121258

[B4] Bailey D. G. Dresser G. K. Leake B. F. Kim R. B. (2007). Naringin is a major and selective clinical inhibitor of organic anion-transporting polypeptide 1A2 (OATP1A2) in grapefruit juice. Clin. Pharmacol. Ther. 81, 495–502. 10.1038/sj.clpt.6100104 17301733

[B5] Balachandran A. Choi S. B. Beata M. M. Małgorzata J. Froemming G. R. A. Lavilla C. A. Jr. (2023). Antioxidant, wound healing potential and *in silico* assessment of naringin, eicosane and octacosane. Molecules 28, 1043. 10.3390/molecules28031043 36770709 PMC9919607

[B6] Barajas-Vega J. L. Raffoul-Orozco A. K. Hernandez-Molina D. Ávila-González A. E. García-Cobian T. A. Rubio-Arellano E. D. (2022). Naringin reduces body weight, plasma lipids and increases adiponectin levels in patients with dyslipidemia. Int. J. Vitam. Nutr. Res. 92, 292–298. 10.1024/0300-9831/a000658 32513069

[B7] Bi C. Jiang Y. Fu T. Hao Y. Zhu X. Lu Y. (2016). Naringin inhibits lipopolysaccharide-induced damage in human umbilical vein endothelial cells via attenuation of inflammation, apoptosis and MAPK pathways. Cytotechnology 68, 1473–1487. 10.1007/s10616-015-9908-3 27006302 PMC4960195

[B8] Capra M. E. Biasucci G. Crivellaro E. Banderali G. Pederiva C. (2023). Dietary intervention for children and adolescents with familial hypercholesterolaemia. Ital. J. Pediatr. 49, 77. 10.1186/s13052-023-01479-8 37349839 PMC10286440

[B9] Chanet A. Milenkovic D. Deval C. Potier M. Constans J. Mazur A. (2012). Naringin, the major grapefruit flavonoid, specifically affects atherosclerosis development in diet-induced hypercholesterolemia in mice. J. Nutr. Biochem. 23, 469–477. 10.1016/j.jnutbio.2011.02.001 21684135

[B10] Choe S. C. Kim H. S. Jeong T. S. Bok S. H. Park Y. B. (2001). Naringin has an antiatherogenic effect with the inhibition of intercellular adhesion molecule-1 in hypercholesterolemic rabbits. J. Cardiovasc. Pharmacol. 38, 947–955. 10.1097/00005344-200112000-00017 11707699

[B11] Dashputre N. L. Laddha U. D. Darekar P. P. Kadam J. D. Patil S. B. Sable R. R. (2023). Potential therapeutic effects of naringin loaded PLGA nanoparticles for the management of alzheimer’s disease: *in vitro*, *ex vivo* and *in vivo* investigation. Heliyon 9, e19374. 10.1016/j.heliyon.2023.e19374 37662728 PMC10474452

[B12] Feng Y. Xu D. (2023). Short-chain fatty acids are potential goalkeepers of atherosclerosis. Front. Pharmacol. 14, 1271001. 10.3389/fphar.2023.1271001 38027009 PMC10679725

[B13] Gao Y. Wang Z. Zhang Y. Liu Y. Wang S. Sun W. (2018). Naringenin inhibits N(G)-nitro-L-arginine methyl ester-induced hypertensive left ventricular hypertrophy by decreasing angiotensin-converting enzyme 1 expression. Exp. Ther. Med. 16, 867–873. 10.3892/etm.2018.6258 30112041 PMC6090443

[B14] Global Cardiovascular Risk Consortium, Magnussen C. Ojeda F. M. Leong D. P. Alegre-Diaz J. Amouyel P. (2023). Global effect of modifiable risk factors on cardiovascular disease and mortality. N. Engl. J. Med. 389, 1273–1285. 10.1056/NEJMoa2206916 37632466 PMC10589462

[B15] Heidary Moghaddam R. Samimi Z. Moradi S. Z. Little P. J. Xu S. Farzaei M. H. (2020). Naringenin and naringin in cardiovascular disease prevention: a preclinical review. Eur. J. Pharmacol. 887, 173535. 10.1016/j.ejphar.2020.173535 32910944

[B16] Hong N. Lin Y. Ye Z. Yang C. Huang Y. Duan Q. (2022). The relationship between dyslipidemia and inflammation among adults in east coast China: a cross-sectional study. Front. Immunol. 13, 937201. 10.3389/fimmu.2022.937201 36032093 PMC9403313

[B17] Hsueh T. P. Sheen J. M. Pang J. H. Bi K. W. Huang C. C. Wu H. T. (2016). The anti-atherosclerotic effect of naringin is associated with reduced expressions of cell adhesion molecules and chemokines through NF-κB pathway. Molecules 21, 195. 10.3390/molecules21020195 26861272 PMC6274007

[B18] Hwang I. C. Kim C. H. Kim J. Y. Choi H. M. Yoon Y. E. Cho G. Y. (2023). Rate of change in 10-year atherosclerotic cardiovascular disease risk and its implications for primary prevention. Hypertension 80, 1697–1706. 10.1161/HYPERTENSIONAHA.122.20678 37470768

[B19] Ikemura M. Sasaki Y. Giddings J. C. Yamamoto J. (2012). Preventive effects of hesperidin, glucosyl hesperidin and naringin on hypertension and cerebral thrombosis in stroke-prone spontaneously hypertensive rats. Phytother. Res. 26, 1272–1277. 10.1002/ptr.3724 22228501

[B20] Jeon S. M. Bok S. H. Jang M. K. Lee M. K. Nam K. T. Park Y. B. (2001). Antioxidative activity of naringin and lovastatin in high cholesterol-fed rabbits. Life Sci. 69, 2855–2866. 10.1016/s0024-3205(01)01363-7 11720089

[B21] Lee E. J. Kim D. I. Kim W. J. Moon S. K. (2009). Naringin inhibits matrix metalloproteinase-9 expression and AKT phosphorylation in tumor necrosis factor-alpha-induced vascular smooth muscle cells. Mol. Nutr. Food Res. 53, 1582–1591. 10.1002/mnfr.200800210 19810018

[B22] Lee E. J. Moon G. S. Choi W. S. Kim W. J. Moon S. K. (2008). Naringin-induced p21WAF1-mediated G(1)-phase cell cycle arrest via activation of the ras/raf/ERK signaling pathway in vascular smooth muscle cells. Food Chem. Toxicol. 46, 3800–3807. 10.1016/j.fct.2008.10.002 18951945

[B23] Li G. Xu Y. Sheng X. Liu H. Guo J. Wang J. (2017). Naringin protects against high glucose-induced human endothelial cell injury via antioxidation and CX3CL1 downregulation. Cell Physiol. biochem. 42, 2540–2551. 10.1159/000480215 28848146

[B24] Li P. Wang S. Guan X. Cen X. Hu C. Peng W. (2014). Six months chronic toxicological evaluation of naringin in Sprague-Dawley rats. Food Chem. Toxicol. 66, 65–75. 10.1016/j.fct.2014.01.023 24462649

[B25] Li P. Wu H. Wang Y. Peng W. Su W. (2020). Toxicological evaluation of naringin: acute, subchronic, and chronic toxicity in Beagle dogs. Regul. Toxicol. Pharmacol. 111, 104580. 10.1016/j.yrtph.2020.104580 31954754

[B26] Li W. Wang C. Peng J. Liang J. Jin Y. Liu Q. (2014). Naringin inhibits TNF-α induced oxidative stress and inflammatory response in HUVECs via Nox4/NF-κ B and PI3K/Akt pathways. Curr. Pharm. Biotechnol. 15 (12), 1173–1182. 10.2174/1389201015666141111114442 25391245

[B27] Liu S. Liu Y. Liu Z. Hu Y. Jiang M. (2023). A review of the signaling pathways of aerobic and anaerobic exercise on atherosclerosis. J. Cell. Physiol. 238, 866–879. 10.1002/jcp.30989 36890781

[B28] Mao Z. Gan C. Zhu J. Ma N. Wu L. Wang L. (2017). Anti-atherosclerotic activities of flavonoids from the flowers of *Helichrysum arenarium* L. MOENCH through the pathway of anti-inflammation. Bioorg. Med. Chem. Lett. 27, 2812–2817. 10.1016/j.bmcl.2017.04.076 28479197

[B29] Memariani Z. Abbas S. Q. Ul Hassan S. S. Ahmadi A. Chabra A. (2021). Naringin and naringenin as anticancer agents and adjuvants in cancer combination therapy: efficacy and molecular mechanisms of action, a comprehensive narrative review. Pharmacol. Res. 171, 105264. 10.1016/j.phrs.2020.105264 33166734

[B30] Oyagbemi A. A. Omobowale T. O. Adejumobi O. A. Owolabi A. M. Ogunpolu B. S. Falayi O. O. (2020). Antihypertensive power of naringenin is mediated via attenuation of mineralocorticoid receptor (MCR)/angiotensin converting enzyme (ACE)/Kidney injury molecule (Kim-1) signaling pathway. Eur. J. Pharmacol. 880, 173142. 10.1016/j.ejphar.2020.173142 32422184

[B31] Parsamanesh N. Moossavi M. Bahrami A. Fereidouni M. Barreto G. Sahebkar A. (2019). NLRP3 inflammasome as a treatment target in atherosclerosis: a focus on statin therapy. Int. Immunopharmacol. 73, 146–155. 10.1016/j.intimp.2019.05.006 31100709

[B32] Pengnet S. Prommaouan S. Sumarithum P. Malakul W. (2019). Naringin reverses high-cholesterol diet-induced vascular dysfunction and oxidative stress in rats via regulating LOX-1 and NADPH oxidase subunit expression. Biomed. Res. Int. 2019, 3708497. 10.1155/2019/3708497 31781614 PMC6855071

[B33] Pi Y. Liang Z. Jiang Q. Chen D. Su Z. Ouyang Y. (2023). The role of PIWI-interacting RNA in naringin pro-angiogenesis by targeting HUVECs. Chem. Biol. Interact. 371, 110344. 10.1016/j.cbi.2023.110344 36623717

[B34] Przybylska S. Tokarczyk G. (2022). Lycopene in the prevention of cardiovascular diseases. Int. J. Mol. Sci. 23, 1957. 10.3390/ijms23041957 35216071 PMC8880080

[B35] Pu P. Gao D. M. Mohamed S. Chen J. Zhang J. Zhou X. Y. (2012). Naringin ameliorates metabolic syndrome by activating AMP-activated protein kinase in mice fed a high-fat diet. Arch. Biochem. Biophys. 518, 61–70. 10.1016/j.abb.2011.11.026 22198281

[B36] Shangguan W. J. Zhang Y. H. Li Z. C. Tang L. M. Shao J. Li H. (2017). Naringin inhibits vascular endothelial cell apoptosis via endoplasmic reticulum stress and mitochondrial mediated pathways and promotes intraosseous angiogenesis in ovariectomized rats. Int. J. Mol. Med. 40, 1741–1749. 10.3892/ijmm.2017.3160 29039439 PMC5716435

[B55] Toth P. P. Patti A. M. Nikolic D. Giglio R. V. Castellino G. Biancucci T. (2015). Bergamot reduces plasma lipids, atherogenic small dense LDL, and subclinical atherosclerosis in subjects with moderate hypercholesterolemia: A 6 months prospective study. Front Pharmacol. 6, 299. 10.3389/fphar.2015.00299 26779019 PMC4702027

[B37] Virani S. S. Alonso A. Aparicio H. J. Benjamin E. J. Bittencourt M. S. Callaway C. W. (2021). Heart disease and stroke statistics-2021 update: a report from the American heart association. Circulation 143, e254–e743. 10.1161/CIR.0000000000000950 33501848 PMC13036842

[B38] Virani S. S. Newby L. K. Arnold S. V. Bittner V. Brewer L. C. Demeter S. H. (2023). 2023 AHA/ACC/ACCP/ASPC/NLA/PCNA guideline for the management of patients with chronic coronary disease: a report of the American heart association/American college of cardiology oint committee on clinical practice guidelines. Circulation 148, e148. 10.1161/cir.0000000000001168 37471501

[B39] Visnagri A. Adil M. Kandhare A. D. Bodhankar S. L. (2015). Effect of naringin on hemodynamic changes and left ventricular function in renal artery occluded renovascular hypertension in rats. J. Pharm. Bioallied Sci. 7, 121–127. 10.4103/0975-7406.154437 25883516 PMC4399010

[B40] Viswanatha G. L. Shylaja H. Moolemath Y. (2017). The beneficial role of naringin-a citrus bioflavonoid, against oxidative stress-induced neurobehavioral disorders and cognitive dysfunction in rodents: a systematic review and meta-analysis. Biomed. Pharmacother. 94, 909–929. 10.1016/j.biopha.2017.07.072 28810519

[B41] Wang F. Zhao C. Tian G. Wei X. Ma Z. Cui J. (2020a). Naringin alleviates atherosclerosis in ApoE−/− mice by regulating cholesterol metabolism involved in gut microbiota remodeling. J. Agric. Food Chem. 68, 12651–12660. 10.1021/acs.jafc.0c05800 33107729

[B42] Wang K. Peng S. Xiong S. Niu A. Xia M. Xiong X. (2020b). Naringin inhibits autophagy mediated by PI3K-Akt-mTOR pathway to ameliorate endothelial cell dysfunction induced by high glucose/high fat stress. Eur. J. Pharmacol. 874, 173003. 10.1016/j.ejphar.2020.173003 32045600

[B43] Wang Y. Li X. Lv H. Sun L. Liu B. Zhang X. (2023). Therapeutic potential of naringin in improving the survival rate of skin flap: a review. Front. Pharmacol. 14, 1128147. 10.3389/fphar.2023.1128147 36937856 PMC10017745

[B44] Wang Z. Wang S. Zhao J. Yu C. Hu Y. Tu Y. (2019). Naringenin ameliorates renovascular hypertensive renal damage by normalizing the balance of renin-angiotensin system components in rats. Int. J. Med. Sci. 16, 644–653. 10.7150/ijms.31075 31217731 PMC6566737

[B45] Wu Y. Cai C. Xiang Y. Zhao H. Lv L. Zeng C. (2021). Naringin ameliorates monocrotaline-induced pulmonary arterial hypertension through endothelial-to-mesenchymal transition inhibition. Front. Pharmacol. 12, 696135. 10.3389/fphar.2021.696135 34335261 PMC8320371

[B46] Xu Z. Zhang M. Li X. Wang Y. Du R. (2022). Exercise ameliorates atherosclerosis via up-regulating serum β-hydroxybutyrate levels. Int. J. Mol. Sci. 23, 3788. 10.3390/ijms23073788 35409148 PMC8998237

[B47] Xulu S. Oroma Owira P. M. (2012). Naringin ameliorates atherogenic dyslipidemia but not hyperglycemia in rats with type 1 diabetes. J. Cardiovasc. Pharmacol. 59, 133–141. 10.1097/FJC.0b013e31823827a4 21964158

[B48] Yntema T. Koonen D. P. Y. Kuipers F. (2023). Emerging roles of gut microbial modulation of bile acid composition in the etiology of cardiovascular diseases. Nutrients 15, 1850. 10.3390/nu15081850 37111068 PMC10141989

[B49] Yu X. Meng X. Yan Y. Wang H. Zhang L. (2022). Extraction of naringin from pomelo and its therapeutic potentials against hyperlipidemia. Molecules 27, 9033. 10.3390/molecules27249033 36558166 PMC9783781

[B50] Yu Y. M. Chao T. Y. Chang W. C. Chang M. J. Lee M. F. (2016). Thymol reduces oxidative stress, aortic intimal thickening, and inflammation-related gene expression in hyperlipidemic rabbits. J. Food Drug Anal. 24, 556–563. 10.1016/j.jfda.2016.02.004 28911561 PMC9336656

[B51] Zhang H. H. Zhou X. J. Zhong Y. S. Ji L. T. Yu W. Y. Fang J. (2022). Naringin suppressed airway inflammation and ameliorated pulmonary endothelial hyperpermeability by upregulating Aquaporin1 in lipopolysaccharide/cigarette smoke-induced mice. Biomed. Pharmacother. 150, 113035. 10.1016/j.biopha.2022.113035 35658207

[B52] Zhao H. Liu M. Liu H. Suo R. Lu C. (2020). Naringin protects endothelial cells from apoptosis and inflammation by regulating the hippo-YAP pathway. Biosci. Rep. 40, BSR20193431. 10.1042/BSR20193431 32091090 PMC7056449

[B53] Zhao H. Zhao J. (2022). Study on the role of naringin in attenuating trimethylamine-N-Oxide-induced human umbilical vein endothelial cell inflammation, oxidative stress, and endothelial dysfunction. Chin. J. Physiol. 65, 217–225. 10.4103/0304-4920.359796 36308076

[B54] Zoubdane N. Abdo R. A. Nguyen M. Bentourkia M. Turcotte E. E. Berrougui H. (2024). High tyrosol and hydroxytyrosol intake reduces arterial inflammation and atherosclerotic lesion microcalcification in healthy older populations. Antioxidants (Basel) 13, 130. 10.3390/antiox13010130 38275655 PMC10812987

